# Systematic review and meta-analysis of the interventional effects of resveratrol in a rat model of myocardial ischemia-reperfusion injury

**DOI:** 10.3389/fphar.2024.1301502

**Published:** 2024-01-19

**Authors:** Dong-Ze Zhang, Ming-Yang Jia, Hong-Yu Wei, Ming Yao, Li-Hong Jiang

**Affiliations:** ^1^ College of Traditional Chinese Medicine, Changchun University of Traditional Chinese Medicine, Changchun, China; ^2^ Department of encephalopathy, Changchun Traditional Chinese Medicine Hospital, Changchun, China; ^3^ Department of Cardiovascular Medicine, Affiliated Hospital of Changchun University of Traditional Chinese Medicine, Changchun, China

**Keywords:** resveratrol, myocardial ischemia-reperfusion, rat animal model, systematic review, meta-analysis

## Abstract

**Objective:** To evaluate the intervention effect of resveratrol on rat model of myocardial ischemia-reperfusion injury.

**Methods:** The relevant studies on the intervention of resveratrol on rat models of myocardial ischemia reperfusion injury were searched in PubMed, Embase, Cochrane Library, Web of Science, China National Knowledge Infrastructure (CNKI), Wanfang and China Science and Technology Journal Database from the start of database establishment to January 2023. Data were extracted from studies that met the inclusion criteria. The results included electrocardiogram (ECG) and myocardial injury markers: ST changes, cardiac troponin I (cTn-I), cardiac troponin T (cTn-T), creatine kinase (CK), creatine kinase-MB (CK-MB) and lactate dehydrogenase (LDH); hemodynamic indicators: heart rate (HR), left ventricular diastolic pressure (LVDP), left ventricular end-diastolic pressure (LVEDP), left ventricular systolic pressure (LVSP), maximum rate of increase of left ventricular pressure (+dp/dtmax), maximum rate of decrease of left ventricular pressure (−dp/dtmax); oxidative damage indicators: nitric oxide (NO), reactive oxygen species (ROS), superoxide dismutase (SOD), malondialdehyde (MDA); inflammatory factors: tumor necrosis factor-α (TNF-α) and interleukin-6 (IL-6); apoptosis index: B-cell lymphoma-2 (Bcl-2), BCL2-Associated X (Bax), cardiomyocyte apoptosis index (AI); heart tissue structure: myocardial infarction size. Finally, a meta-analysis of these results was conducted. The methodological quality of the studies was assessed using the SYRCLE Bias Risk tool.

**Results:** A total of 43 studies were included in the meta-analysis, and the quality of the included studies was assessed. It was found that the evidence quality of these 43 studies was low, and no study was judged to have low risk bias in all risk assessments. The results showed that resveratrol could reduce ST segment, cTn-I, cTn-T, CK, CK-MB, LDH, LVEDP, ROS, MDA, TNF-α, IL-6, AI levels and myocardial infarction size. HR, LVDP, LVSP, +dp/dtmax, NO, Bcl-2, and SOD levels were increased. However, resveratrol had no significant effect on -dp/dtmax and Bax outcome measures.

**Conclusion:** Resveratrol can reduce ST segment in rat model of myocardial ischemia-reperfusion injury, alleviate myocardial injury, improve ventricular systolic and diastolic ability in hemodynamics, reduce inflammatory response and oxidative damage, and reduce myocardial necrosis and apoptosis. Due to the low quality of the methodologies included in the studies, additional research is required.

## 1 Introduction

Acute myocardial infarction (AMI) is the leading cause of heart failure and cardiogenic death in clinical patients, early treatment of AMI focuses on restoring blood flow to the ischemic danger zone to minimize irreversible tissue damage (Cadenas, 2018). Revascularization and early reperfusion through thrombolysis, coronary artery bypass, or percutaneous coronary interventional therapy to restore blood flow, reduce necrosis, and reduce infarct size have been the main methods for the treatment of patients with acute myocardial infarction (Ibanez et al., 2018; Al et al., 2019). Although early reperfusion is crucial for saving myocardial injury, the reperfusion process itself is a double-edged sword. A series of damaging changes, such as myocardial ultrastructure, energy metabolism, cardiac function and electrophysiology, often cause additional myocardial injury. Increased complications and mortality after cardiac perfusion (Braunwald and Kloner, 1985). Reperfusion injury is an extremely complex pathological process, which may lead to a series of pathological reactions including oxidative stress (Li et al., 2023), calcium overload (Li et al., 2021), inflammation (Algoet et al., 2023), autophagy (Xing et al., 2022; Popov et al., 2023) and apoptosis (Zeng et al., 2019). These pathological reactions are exactly the starting points and important mechanisms for research.

Resveratrol (3,4,5-trihydroxy-trans-stilbene, RES) is a naturally occurring phytoalexin, a member of the stilbene family of phenolic compounds isolated from the roots of white hellebore and polygonum cuspidatum widely used traditional Chinese medicine (Brouwer et al., 2018). In addition, grapes, wine, mulberries, cranberries, and peanuts are known to contain resveratrol in different concentrations (de Freitas et al., 2018; Fu et al., 2018). Researchs showed that resveratrol can protect nervous system, renal, liver, intestine and other organs and tissues from ischemia-reperfusion (Parlar and Arslan, 2019; Wang et al., 2020; Sarkaki et al., 2021; Totonchi et al., 2022). So far, resveratrol has been used to treat polycystic ovary syndrome, cancer, diabetes and other diseases (Jeyaraman et al., 2020; Ren et al., 2021; Ali et al., 2023). In recent years, many animal experiments have confirmed that resveratrol can participate in the protection of cardiovascular system through various physiological mechanisms such as anti-oxidation, anti-apoptosis, anti-autophagy and anti-aging (Feng et al., 2020; Li et al., 2022; Tian et al., 2023). Therefore, we conducted a comprehensive systematic review and meta-analysis of the protective effects and mechanisms of resveratrol on myocardial ischemia-reperfusion injury (MIRI) in rat studies, which only included relevant data from rats to avoid heterogeneity caused by rats and other animal species. In order to provide reference and evidence-based medical evidence for clinical treatment.

## 2 Data and methods

### 2.1 Search strategy

We scientifically searched the PubMed, Embase, Cochrane Library, China National Knowledge Infrastructure (CNKI), China Science and Technology Journal Database, Wanfang, Web of Science databases for resveratrol in rats model on myocardial I/R injury. The search period was from the establishment of each database to January 2023. Subject words + free words were used for retrieval, included “Resveratrol,” “Myocardial Reperfusion Injury” AND “Rats”. Each subject word was connected with its following free words by OR, and then these three parts were connected by AND (Prill et al., 2021).

### 2.2 Inclusion and exclusion criteria

Studies that met the following inclusion criteria were included: 1) Study object: rat myocardial ischemia-reperfusion injury model, the model preparation method must be approved; 2) Intervention measures: the drug used must be resveratrol or a preparation of it, the experimental group cannot be combined with other drugs, and the control group did not use any drugs or only used placebo intervention; 3) Outcome indicators: Indicators evaluating drug effectiveness must be reported in numerical form to ensure that key indicators, including their mean and standard deviation, can be extracted or calculated directly or indirectly; 4) Literature type: Chinese or English literature.

The exclusion criteria were as follows: 1) Research involved laboratory animals other than rats; 2) The experimental group or control group involved the use of other drugs; 3) The data of the evaluation indicators were incomplete or in non-numerical form; 4) The types of article were review, meta-analysis, systematic review or conference report; 5) Non-Chinese and English literature.

### 2.3 Data extraction

After literature retrieval, references that were duplicated, non-full-text, and did not meet the inclusion criteria were excluded, and the following information was extracted from the references that met the inclusion criteria: first author, publication year, experimental animal category, modeling method, number of models, administration route、dose and time, numerical detection indexes, and extractable results. Data extraction was carried out by two researchers (ZDZ and JMY) independently, the average data error of two reviewers should be controlled within 1%. Any discrepancies during the process were resolved by discussion and consensus, then the data were checked by another two reviewers (WHY and JLH). If the disagreement was not resolved, a third investigator (YM) extracted the data again and made the final decision. For important data which cannot be extracted, we can send an email to the author to obtain if necessary. Finally, the outcome indicators included in the meta-analysis were summarized.

### 2.4 Quality assessment

SYRCLE bias risk tool was used to evaluate the methodological quality of the included studies, which was divided into five parts: selection bias, implementation bias, measurement bias, loss of access bias and reporting bias. The evaluation results were finally expressed as “+,” “−” and “?,” included among these “+” represented “low risk bias” and “−” represented “high risk bias,” “?” stands for “uncertain risk bias”. SYRCLE tool is based on the development method of Cochrane bias risk assessment tool (Hooijmans et al., 2014). It is a tool for objective evaluation of possible bias or confounding in the design, implementation and measurement of animal experiments. It is suitable for different types and fields of animal experiments.

### 2.5 Statistical analysis

Outcomes were pooled if they were reported in at least three studies. Review Manager 5.3 software was used for statistical analysis. The quantitative data and the continuous variables were expressed as mean difference (MD) or standardized mean difference (SMD), 95% confidence interval (95% CI) was used for interval estimation. The heterogeneity of the included studies was assessed using the I^2^ test. If *p* < 0.1 or I^2^ > 50%, it indicates that there is a large heterogeneity, and then the random effects model is used for combined analysis. On the contrary, the fixed effect model is used for analysis. When *p* < 0.05, the difference between the two groups was considered statistically significant.

## 3 Results

### 3.1 Results of the literature review

**FIGURE 1:**
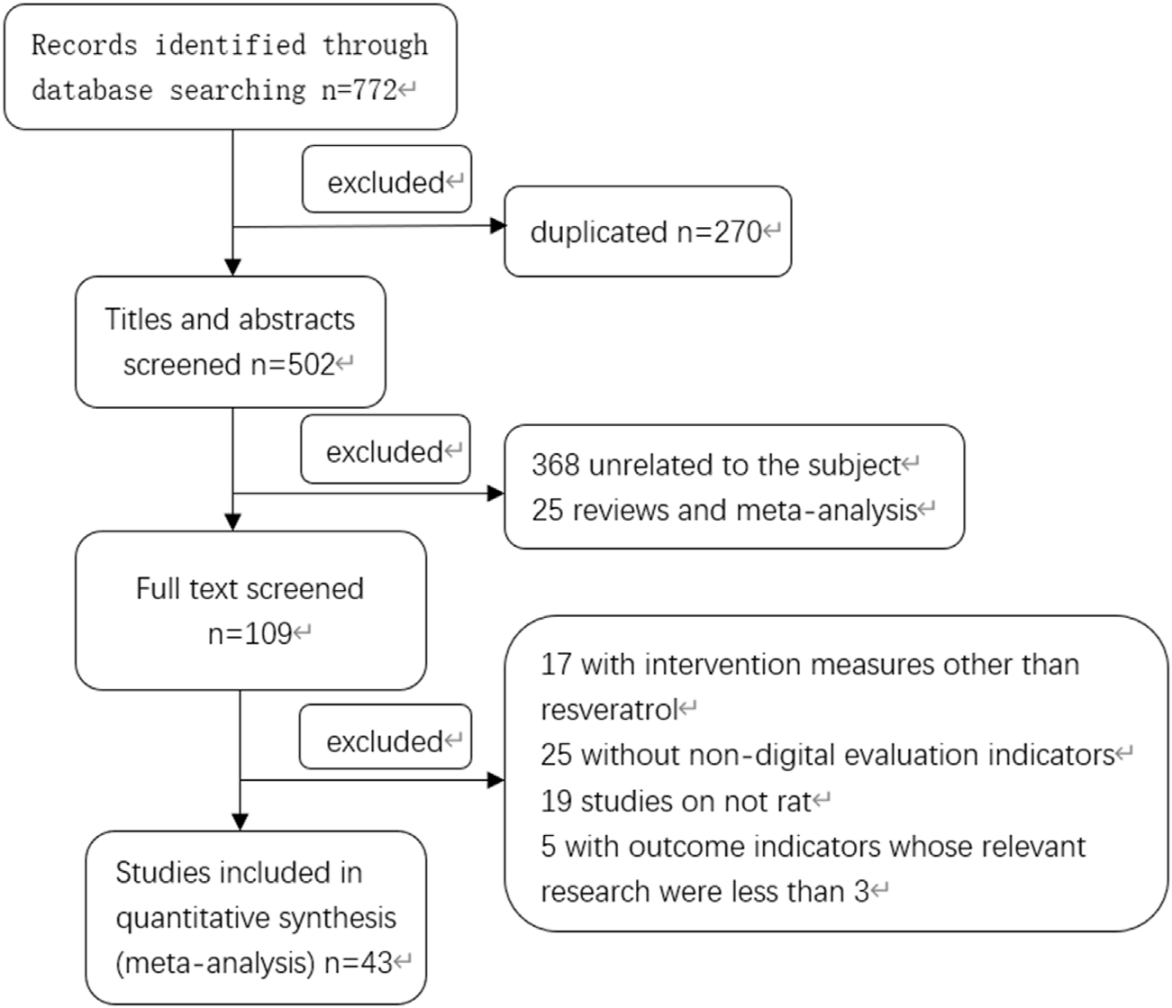
Document retrieval flow chart.

According to the search strategy, a total of 772 Chinese and English literatures were retrieved from PubMed and other databases. According to the inclusion and exclusion criteria, a total of 43 literatures meeting the requirements were screened after eliminating duplicate literatures, reading titles, abstracts and full texts (Dernek et al., 2004; Huang et al., 2004; Das et al., 2005; Chen, 2006; Das et al., 2006; Shen et al., 2006; Wang et al., 2006; Jiang et al., 2007; Xiao et al., 2007; Zhang et al., 2007; Huang et al., 2009; Liu, 2011; Wu et al., 2011; Yang and Zhou, 2011; Dong, 2012; He et al., 2012; He et al., 2013; Ta et al., 2013; Wang et al., 2014; Song et al., 2015; Wang and Wang, 2015; Wu and Liu, 2015; Yang et al., 2016; Song et al., 2017; Wu et al., 2017; Deng et al., 2018; Li et al., 2018; Wang and Zhang, 2018; He et al., 2019; Hu et al., 2019; Qian et al., 2019; Xue et al., 2019; Zhang et al., 2019; Xu et al., 2020a; Bing et al., 2020; Xu et al., 2020b; Fu, 2020; Kazemirad and Kazerani, 2020; Zhao et al., 2020; Yang, 2021; Ma, 2022; Wang et al., 2022; Yan et al., 2022), and the specific screening process was shown in Figure 1. There were a total of 974 rat animal models, including 487 in the control group and 487 in the experimental group. The basic information included in the study is shown in Table 1.

**TABLE 1:** Description of the characteristics of studies included in this review.

Study ID	Animals	Modeling method	No. Of animals (control group/resveratrol group)	Administration route	Dosage	Drug administration time	Outcome indexes
Wang et al. (2014)	Male SD rats (200–250 g)	Langendorff, ischemia30 min, reperfusion40 min	12/12	ecp	15 mg/L	perfusion period	HR,LVDP, LVEDP,+dp/dtmax,-dp/dtmax,CK,LDH
Qian et al. (2019)	Male Wistar rats (200–250 g)	Ligature LCA30 min, restore blood supply	12/12	i.g	50 mg/kg/d	7 days before surgery	ST,+dp/dtmax,-dp/dtmax,CK-MB; LDH,MDA,SOD,cTn-I
Jiang et al. (2007)	Male SD rats (180–240 g)	Ligature LAD30 min, reperfusion30 min	10/10	i.v	20 mg/kg	pre-ligation	ST,LDH,SOD,MDA,Bcl-2,BAX
Hu et al. (2019)	Male SD rats (200–220 g)	Ligature LAD30 min, restore blood supply	8/8	i.p	15 mg/kg/d	7 days before surgery	CK-MB,LDH,TNF-α,IL-6
Huang et al. (2004)	Male SD rats (220–240 g)	Ligature LAD40 min, reperfusion120 min	8/8	i.v	2.5/5/10 mg/kg	1 min before ligation and reperfusion	HR,ST, MI size,CK,LDH
Bing et al. (2020)	Male SD rats (180–230 g)	Ligature LAD30 min, reperfusion120 min	15/15	i.p	30 mg/kg	45min before surgery	MI size
Song et al. (2015)	Male SD rats (240–260 g)	Ligature LAD35 min, reperfusion120 min	10/10	i.v	1 mL/kg	15min before ligation, 1minbefore reperfusion	MDA,SOD,CK-MB
He et al. (2019)	Male SD rats (200–220 g)	Ligature LAD30 min, reperfusion120 min	10/10	i.p	15 mg/kg/d	5 days before surgery	CK-MB,LDH,TNF-α,IL-6,SOD,MDA
Wang and Wang (2015)	Rats (230–240 g)	Ligature LAD40 min, reperfusion120 min	9/9	i.v	10 mg/kg	1 min before ligation and reperfusion	ST,CK,LDH,MDA,SOD
Huang et al. (2009)	Male SD rats (220–240 g)	Ligature LAD40 min, reperfusion120 min	8/8	i.v	2.5/5/10 mg/kg	1 min before ligation and reperfusion	ST, MI size,CK,LDH,SOD,MDA
Wang and Zhang (2018)	Male SD rats (220–250 g)	Ligature LAD40 min, reperfusion 120 min	10/10	i.v	10 mg/kg	15min before surgery	LDH,CK,cTn-I,LVSP, LVEDP,+dp/dtmax,-dp/dtmax
Zhang et al. (2019)	Male SD rats (220–250 g)	Ligature LAD45 min, reperfusion120 min	15/15	i.v	20 mg/kg	1 min before ligation	cTn-T,CK-MB,Bcl-2,BAX,AI
Xu et al. (2020a)	Male SD rats (200–220 g)	Ligature LAD30 min, restore blood supply	10/10	i.p	15 mg/kg/d	7 days before surgery	CK-MB,LDH,MDA,ROS,SOD
Liu (2011)	Male Wistar rats (180–195 g)	Langendorff, ischemia45 min,reperfusion120 min	6/6	ecp	10umol/L	perfusion period	HR,LVSP, LVEDP,+dp/dtmax,CK-MB, MI size
Chen (2006)	Male SD rats (270–300 g)	Ligature LCA30 min, reperfusion120 min	10/10	i.v	10 mg/kg	15min before ligation, 1min before reperfusion	LVSP,+dp/dtmax,-dp/dtmax,MDA,SOD, AI
Xiao et al. (2007)	Male Wistar rats (190–210 g)	Langendorff, ischemia20 min, reperfusion30 min	8/8	ecp	30umol/L	perfusion period	SOD,MDA
Dong (2012)	Male SD rats (200–220 g)	Ligature LAD30 min, reperfusion120 min	10/10	i.v	2.5/5/10 mg/kg	15min before ligation	MI size,cTn-T,CK-MB
Shen et al. (2006)	Male SD rats (250–300 g)	Ligature LCA30 min, reperfusion120 min	15/15	i.v	10 mg/kg	15min before ligation	NO,MDA
Yan et al. (2022)	Male SD rats (200 ± 10 g)	Ligature LAD40 min, reperfusion120 min	10/10	i.g	10/20/30 mg/kg/d	7 days before surgery	CK-MB,LDH,SOD,MDA
Xue et al. (2019)	Male SD rats (280–300 g)	Langendorff, ischemia30 min, reperfusion60 min	15/15	ecp	40 μmol/L	perfusion period	LVDP,HR,+dp/dtmax,-dp/dtmax,AI, MI size,SOD,MDA
Wang et al. (2022)	Male SD rats (200 ± 20 g)	Ligature LAD30 min, reperfusion120 min	15/15	i.v	20 mg/kg	15 min before ligation, 1 min before reperfusion	LVSP, LVEDP,+dp/dtmm,-dp/dtmm,CK-MB,LDH,TNF-α,IL-6
Zhao et al. (2020)	Male SD rats (220–280 g)	Ligature LCA30min, restore blood supply	15/15	i.p	15 mg/kg/d	7 days before surgery	CK-MB,LDH,MDA,SOD, MI size
Ma (2022)	Male SD rats (220–280 g)	Ligature LAD30 min, reperfusion120 min	10/10	i.p	20 mg/kg/d	7 days before surgery	CK-MB,LDH, MI size,SOD,ROS
Fu (2020)	Male SD rats (240–280 g)	Ligature LAD30min, reperfusion120min	10/10	i.p	20 mg/kg/d	7 days before surgery	CK-MB,LDH,MDA,SOD,TNF-α,IL-6
Deng et al. (2018)	Male SD rats (200–300 g)	Ligature LAD30 min, reperfusion120 min	15/15	i.v	20 mg/kg	1 min before reperfusion	MDA,SOD
Yang (2021)	Male SD rats (180–200 g)	Ligature LAD30 min, reperfusion120 min	10/10	i.g	7.5 mg/kg/d	7 days before surgery	LDH,CK-MB,cTn-I,cTn-T, MI size,ROS,MDA,SOD
Li et al. (2018)	Male SD rats (200–300 g)	Ligature LAD30min, reperfusion120min	20/20	i.v	20 mg/kg	1 min before reperfusion	MDA,SOD
Song et al. (2017)	Male SD rats (220 ± 30 g)	Ligature LAD20 min, reperfusion40 min	10/10	i.v	1 mL/kg	15 min before ligation, 1 minbefore reperfusion	LVSP,+dp/dtmax,-dp/dtmax
Wang et al. (2006)	Male SD rats (250–275 g)	Langendorff, ischemia 30 min, reperfusion120 min	14/14	ecp	10umol/L	perfusion period	NO,MDA,LVDP,+dp/dtmax,-dp/dtmax, MI size,AI
Yang and Zhou (2011)	SD rats (250–300 g)	Langendorff, ischemia30 min, reperfusion120 min	8/8	ecp	10umol/L	perfusion period	LVDP,+dp/dtmax,-dp/dtmax
Wu et al. (2017)	Male SD rats (240–260 g)	Ligature LAD35 min, reperfusion120 min	10/10	i.v	10 mg/kg	15min before ligation, 1min before reperfusion	NO
Xu et al. (2020b)	Male SD rats (220–280 g)	Ligature LAD30 min, reperfusion120 min	15/15	i.p	15 mg/kg/d	7 days before surgery	HR, MI size,CK-MB,LDH,SOD,MDA
Ta et al. (2013)	Male SD rats (250–300 g)	Ligature LAD5 min, reperfusion5 min, repeat four times	28/28	i.g	20 mg/kg/d	7 days before surgery	MI size,AI
He et al. (2013)	Male SD rats (220–250 g)	Ligature LAD45min, reperfusion120min	10/10	i.v	20 mg/kg	1 min before ligation and reperfusion	LVSP, LVEDP,+dp/dtmax,-dp/dtmax
Wu and Liu (2015)	Male SD rats (200–250 g)	Ligature LAD30 min, reperfusion120 min	12/12	i.v	10 mg/kg	15min before ligation, 1minbefore reperfusion	LDH; CK-MB
Das et al. (2005)	Male SD rats (275–300 g)	Langendorff, ischemia30 min, reperfusion120 min	6/6	i.g	2.5 mg/kg/d	7 days before surgery	HR,LVDP, MI size,AI
Dernek et al. (2004)	Male SD rats (250–300 g)	Langendorff, ischemia60 min, reperfusion60 min	10/10	i.g	20 mg/kg/d	14 days before surgery	+dp/dtmax,LDH,CK-MB,cTn-I
Kazemirad and Kazerani (2020)	Male Wistar rats (250–300 g)	Langendorff, ischemia30 min, reperfusion120 min	10/10	ecp	10umol/L	perfusion period	CK-MB,LDH,HR,LVDP,+dp/dtmax
Das et al. (2006)	Male SD rats (250–300 g)	Langendorff, ischemia30 min, reperfusion120min	6/6	ecp	10umol/L	perfusion period	LVDP, MI size,MDA
Yang et al. (2016)	Male SD rats (250–300 g)	Langendorff, ischemia30min, reperfusion60 min	10/10	ecp	10umol/L	perfusion period	MI size,AI
Zhang et al. (2007)	half male and half female SD rats (270 ± 15 g)	Ligature LCA30 min, reperfusion120 min	8/8	i.g	10/20/40 mg/kg/d	7 days before surgery	ST, MI size,CK,LDH,MDA,SOD
He et al. (2012)	Male SD rats (220–250 g)	Ligature LAD45 min, reperfusion120 min	10/10	i.v	20 mg/kg	1 min before ligation	NO,Bcl-2,Bax
Wu et al. (2011)	Male Wistar rats (240–270 g)	Ligature LAD45 min, restore blood supply	12/12	i.v	10 mg/kg	15 min before ligation, 1 minbefore reperfusion	SOD,CK,LDH,Bcl-2,Bax

Note: i.g: Intragastric administration i.p: intraperitoneal injection i.v: intravenous injection ecp: extracorporeal perfusion; SD, Sprague-Dawley; NO, nitric oxide;MDA, malondialdehyde; LVDP, left ventricular diastolic pressure; +dp/dtmax, maximum rate of increase of left ventricular pressure; -dp/dtmax, maximum rate of decrease of left ventricular pressure; MI, size; AI, cardiomyocyte apoptosis index; LVDP, left ventricular diastolic pressure; HR, heart rate; CK-MB, creatine kinase-MB; LDH, lactate dehydrogenase; SOD, superoxide dismutase; LVSP, left ventricular systolic pressure; LVEDP, left ventricular end-diastolic pressure; ,cTn-I, cardiac troponin I; Bcl-2,B-cell lymphoma-2; Bax. BCL2-Associated X; TNF-α, tumor necrosis factor-α; IL-6, interleukin-6; cTn-T, cardiac troponin T; CK, creatine kinase; LAD, left anterior descending coronary artery; LCA, left coronary artery; min,minute/minutes; No,number.

### 3.2 Methodological quality evaluation

The SYRCLE bias risk tool was used to assess the methodological quality of the included studies, and the specific assessment method was interpreted according to the example of Gong-cai Tao et al. (Tao et al., 2019). The results are shown in Table 2. Fourteen studies (32.6%) described methods for generating random sequences, such as random assignment of random number tables (Chen, 2006; Yang and Zhou, 2011; Song et al., 2015; Wang and Wang, 2015; Wu et al., 2017; Xu and; He et al., 2019; Hu et al., 2019; Qian et al., 2019; Fu, 2020; Zhao et al., 2020; Zhao et al., 2020; Yang, 2021; Ma, 2022; Wang et al., 2022). Randomization of animal placement was reported in 17 studies (39.5%) (Dernek et al., 2004; Chen, 2006; Das et al., 2006; Wang et al., 2014; Yang et al., 2016; Deng et al., 2018; Li et al., 2018; Wang and Zhang, 2018; He et al., 2019; Xu et al., 2020a; Bing et al., 2020; Xu et al., 2020b; Fu, 2020; Zhao et al., 2020; Ma, 2022; Wang et al., 2022; Yan et al., 2022). All 43 studies included reported selective results. Baseline characteristics, hidden grouping, blind method (breeder + researcher) are all uncertainty risk bias. In addition, incomplete data were reported in two studies (4.7%) (Song et al., 2015; Bing et al., 2020). In the randomness outcome assessment, three studies (Wu et al., 2011; Wang et al., 2014; Xue et al., 2019) were evaluated with uncertainty risk bias, and the rest were all high-risk bias. All studies are registered without a pre-specified protocol.

**TABLE 2:** SYRCLE’s RoB tool for each experimental animal studies.

Study ID	1	2	3	4	5	6	7	8	9
	Selection bias			Performance bias		Measurement bias		Withdraw bias	Reporting bias
Wang et al. (2014)	?	?	?	+	?	?	?	?	+
Qian et al. (2019)	+	?	?	-	?	-	-	?	+
Jiang et al. (2007)	?	?	?	-	?	-	?	?	+
Hu et al. (2019)	+	?	?	?	?	-	-	?	+
Huang et al. (2004)	?	?	?	-	?	-	-	?	+
Bing et al. (2020)	?	?	?	+	?	-	?	-	+
Song et al. (2015)	+	?	?	-	?	-	-	-	+
He et al. (2019)	+	?	?	+	?	-	?	?	+
Wang and Wang (2015)	+	?	?	-	?	-	?	?	+
Huang et al. (2009)	?	?	?	-	?	-	?	?	+
Wang and Zhang (2018)	?	?	?	+	?	-	?	?	+
Zhang et al. (2019)	?	?	?	-	?	-	?	?	+
Xu et al. (2020a)	+	?	?	+	?	-	?	?	+
Liu (2011)	?	?	?	-	?	-	?	?	+
Chen (2006)	+	?	?	+	?	-	?	?	+

Study ID	1	2	3	4	5	6	7	8	9
	Selection bias			Performance bias		Measurement bias		Withdraw bias	Reporting bias
Xiao et al. (2007)	?	?	?	-	?	-	?	?	+
Dong (2012)	?	?	?	-	?	-	?	?	+
Shen et al. (2006)	?	?	?	-	?	-	?	?	+
Yan et al. (2022)	?	?	?	+	?	-	?	?	+
Xue et al. (2019)	?	?	?	-	?	?	?	?	+
Wang et al. (2022)	+	?	?	+	?	-	?	?	+
Zhao et al. (2020)	+	?	?	+	?	-	?	?	+
Ma (2022)	?	?	?	+	?	-	?	?	+
Fu (2020)	+	?	?	+	?	-	?	?	+
Deng et al. (2018)	?	?	?	+	?	-	?	?	+
Yang (2021)	+	?	?	?	?	-	?	?	+
Li et al. (2018)	?	?	?	+	?	-	?	?	+
Song et al. (2017)	?	?	?	-	?	-	?	?	+
Zhang et al. (2007)	?	?	?	-	?	-	?	?	+
He et al. (2012)	?	?	?	-	?	-	?	?	+
Wu et al. (2011)	?	?	?	-	?	?	?	?	+
Wang et al. (2006)	?	?	?	-	?	-	?	?	+
Yang and Zhou (2011)	+	?	?	-	?	-	-	?	+
Wu et al. (2017)	+	?	?	-	?	-	-	?	+
Xu et al. (2020b)	+	?	?	+	?	-	?	?	+
Ta et al. (2013)	?	?	?	-	?	-	-	?	+
He et al. (2013)	?	?	?	-	?	-	-	?	+
Wu and Liu (2015)	?	?	?	-	?	-	?	?	+
Das et al. (2005)	-	?	?	-	?	-	-	?	+
Dernek et al. (2004)	-	?	?	+	?	-	-	?	+
Kazemirad and Kazerani (2020)	-	?	?	?	?	-	-	?	+
Das et al. (2006)	-	?	?	+	?	-	?	?	+
Yang et al. (2016)	?	?	?	+	?	-	-	?	+

Note1: +: low risk of bias -: high risk of bias ?: uncertain risk of bias Note2:1: Sequence generation 2: baseline characteristics 3: Hidden grouping 4: Animal placement randomization 5: Blind method (Breeders + researchers) 6: Stochastic result evaluation 7: Blind (outcome evaluator) 8: Incomplete data report 9: Selective result reporting.

### 3.3 Results of six outcome indicators and subgroup analyses

By summarizing the included outcome indicators, the effects of resveratrol on MIRI in rats could be classified to six aspects: electrocardiogram changes and myocardial injury markers, hemodynamics, oxidative damage, inflammatory factors, apoptosis, cardiac tissue structure. As followed: 1) ECG changes and markers of myocardial injury: ST segment changes, creatine kinase (CK), creatine kinase-MB (CK-MB), cardiac troponin T (cTn-T), cardiac troponin I (cTn-I), lactate dehydrogenase (LDH); 2) Hemodynamics: heart rate (HR), left ventricular diastolic pressure (LVDP), left ventricular end-diastolic pressure (LVEDP), maximum rate of increase of left ventricular pressure (+dp/dtmax), maximum rate of decrease of left ventricular pressure (−dp/dtmax), left ventricular systolic pressure (LVSP); 3) Oxidative damage: malondialdehyde (MDA), superoxide dismutase (SOD), nitric oxide (NO), reactive oxygen species (ROS); 4) Inflammatory factors: Interleukin-6 (IL-6), tumor necrosis factor-α (TNF-α); 5) Apoptosis: cardiomyocyte apoptosis index (AI), B-cell lymphoma-2 (Bcl-2), BCL2-Associated X (Bax); 6) Heart tissue structure: myocardial infarction size.

#### 3.3.1 Effects of resveratrol on ST segment and markers of myocardial injury in myocardial ischemia-reperfusion injury model in rats

**FIGURE 2:**
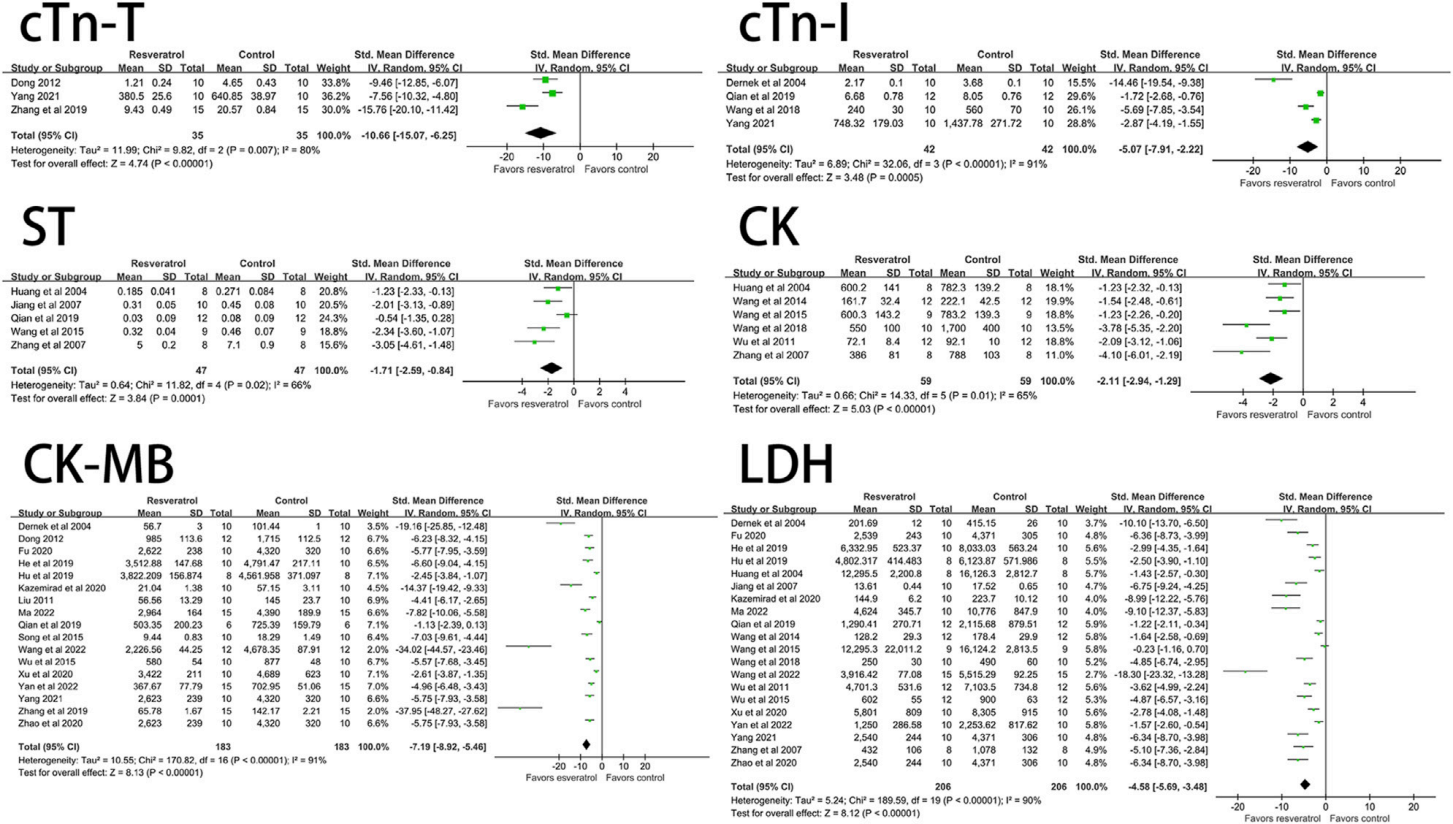
Forest plot of effects of resveratrol on CK, CK-MB, cTn-T, cTn-I, ST, LDH in rats with myocardial ische mia-reperfusion injury. CK, creatine kinase; CK-MB, creatine kinase-MB; cTn-T, cardiac troponin T; cTn-I, cardiac troponin I; LDH, lactate dehydrogenase.

The effects of resveratrol on ST segment change, CK, CK-MB, LDH, cTn-T, cTn-I on myocardial ischemia-reperfusion injury model in rats were analyzed. The results showed that the experimental group (resveratrol) could significantly reduce the elevation of ST segment (five studies, 94 animal models, I^2^ = 66%, *p* < 0.05, SMD = −1.71 [−2.59, −0.84]) and CK (six studies, 118 animal models, I^2^ = 65%, *p* < 0.05 SMD = −2.11 [−2.94, −1.29]) compared with the control group. CK-MB (17 studies, 356 animal models, I^2^ = 91%, *p* < 0.05, SMD = −7.19 [−8.92, −5.46]), LDH (20 studies, 412 animal models, I^2^ = 90%, *p* < 0.05, SMD = −4.58 [−5.69, −3.48]), cTn-T (three studies, 70 animal models, I^2^ = 80%, *p* < 0.05, SMD = −10.66 [-15.07, −6.25]), cTn-I (four studies, 84 animal models, I^2^ = 91%, *p* < 0.05, SMD = −5.07 [−7.91, −2.22]) level of outcome indicator. The forest plot of meta-analysis is shown in Figure 2.

#### 3.3.2 Effect of resveratrol on hemodynamics of myocardial ischemia-reperfusion injury model in rats

**FIGURE 3:**
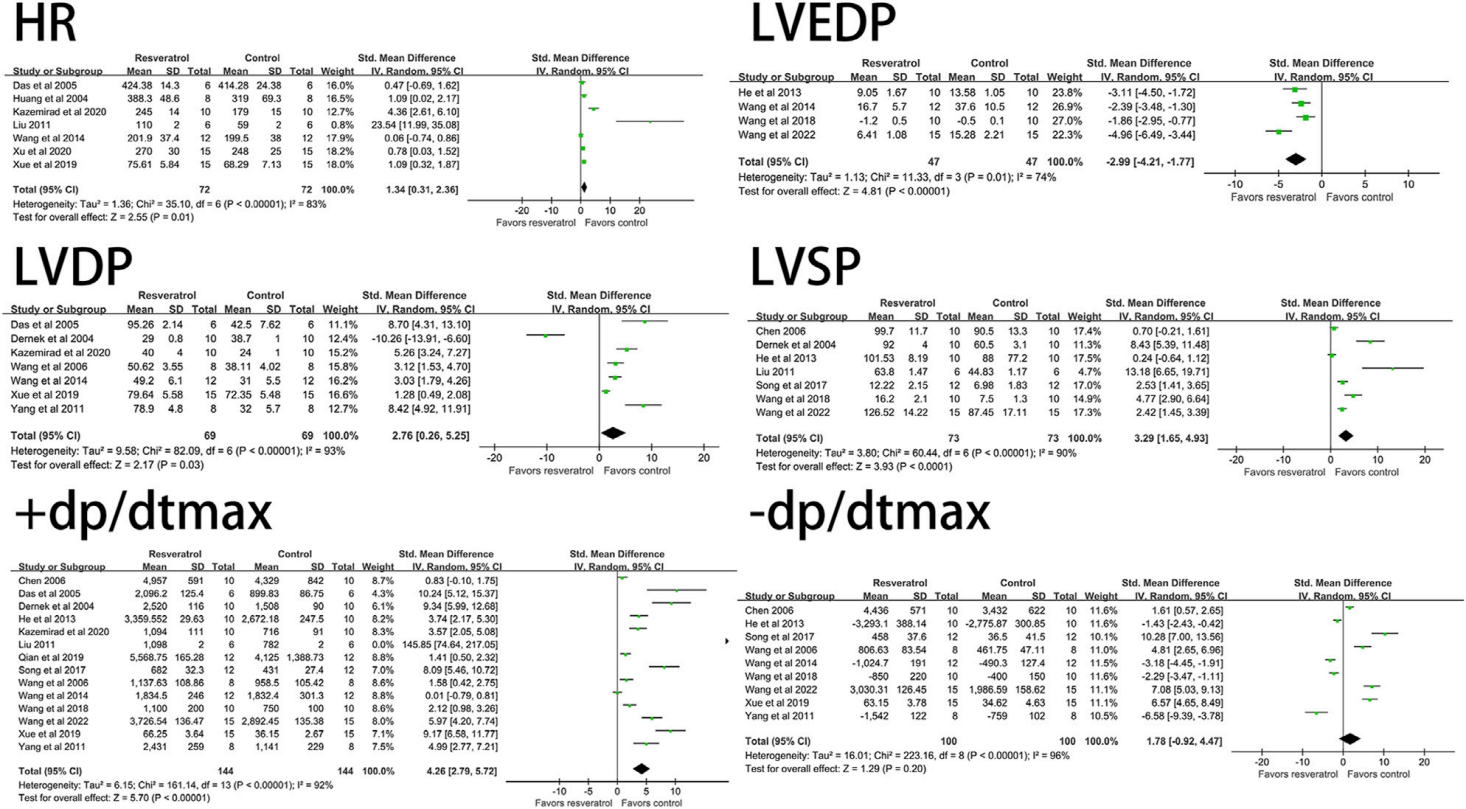
Forest plot of effects of resveratrol on HR, LVEDP, LVDP, LVSP, +dp/dtmax, -dp/dtmax in rats with myocardial ischemia-reperfusion injury. HR, heart rate; LVEDP, Ieft ventricular end-diastolic pressure; LVDP, Ieft ventricular diastolic pressure; LVSP, left ventricular systolic pressure; +dp/dtmax, maximum rate of increase of left ventricular pressure; -dp/dtmax, maximum rate of decrease of left ventricular pressure.

Resveratrol significantly decreased the level of LVEDP (four studies, 94 animal models, I^2^ = 74%, *p* < 0.05, SMD = −2.99 [−4.21, −1.77]) and significantly increased LVDP (seven studies, 138 animal models, I^2^ = 93%, *p* < 0.05). SMD = 2.76 [0.26, 5.25]), LVSP (seven studies, 146 animal models, I^2^ = 90%, *p* < 0.05, SMD = 3.29 [1.65, 4.93]), +dp/dtmax (14 studies, 288 animal models, I^2^ = 92%, *p* < 0.05, SMD = 4.26 [2.79, 5.72]), HR (seven studies, 144animal models, I^2^ = 83%, *p* < 0.05, SMD = 1.34 [0.31, 2.36]) levels. There was no significant effect on -dp/dtmax (nine studies, 200 animal models, I^2^ = 96%, *p* = 0.20, SMD = 1.78 [−0.92, 4.47]). See Figure 3 for forest plot.

#### 3.3.3 Effects of resveratrol on oxidative damage in rat model of myocardial ischemia-reperfusion injury

**FIGURE 4:**
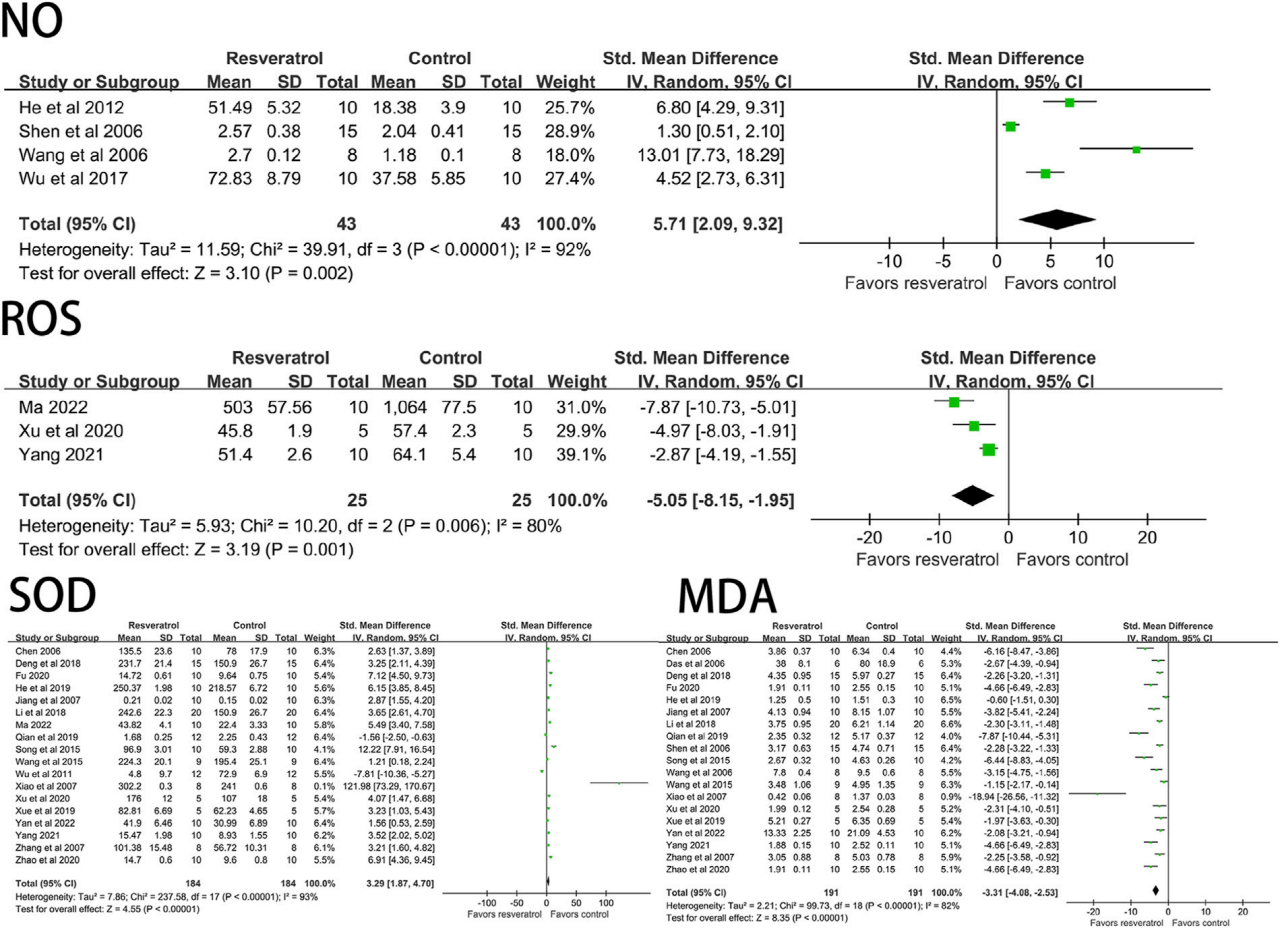
Forest plot of effects of resveratrol on NO, ROS, SOD, MDA in rats with myocardial ischemia-reperfusion injury. NO, nitric oxide; ROS, reactive oxygen species; SOD, superoxide dismutase; MDA, malondialdehyde.

Resveratrol significantly decreased ROS (three studies, 50 animal models, I^2^ = 80%, *p* < 0.05, SMD = −5.05 [−8.15, −1.95]), MDA (19 studies, 382 animal models, I^2^ = 82%, *p* < 0.05, SMD = −3.31 [−4.08, −2.53]), but significantly increased the level of NO (four studies, 86 animal models, I^2^ = 92%, *p* < 0.05, SMD = 5.71 [2.09, 9.32]) and SOD (18 studies, 368 animal models, I^2^ = 93%, *p* < 0.05, SMD = 3.29 [1.87, 4.70]) value. See Figure 4 for forest plot.

#### 3.3.4 Effects of resveratrol on inflammatory factors in rat model of myocardial ischemia-reperfusion injury

**FIGURE 5:**
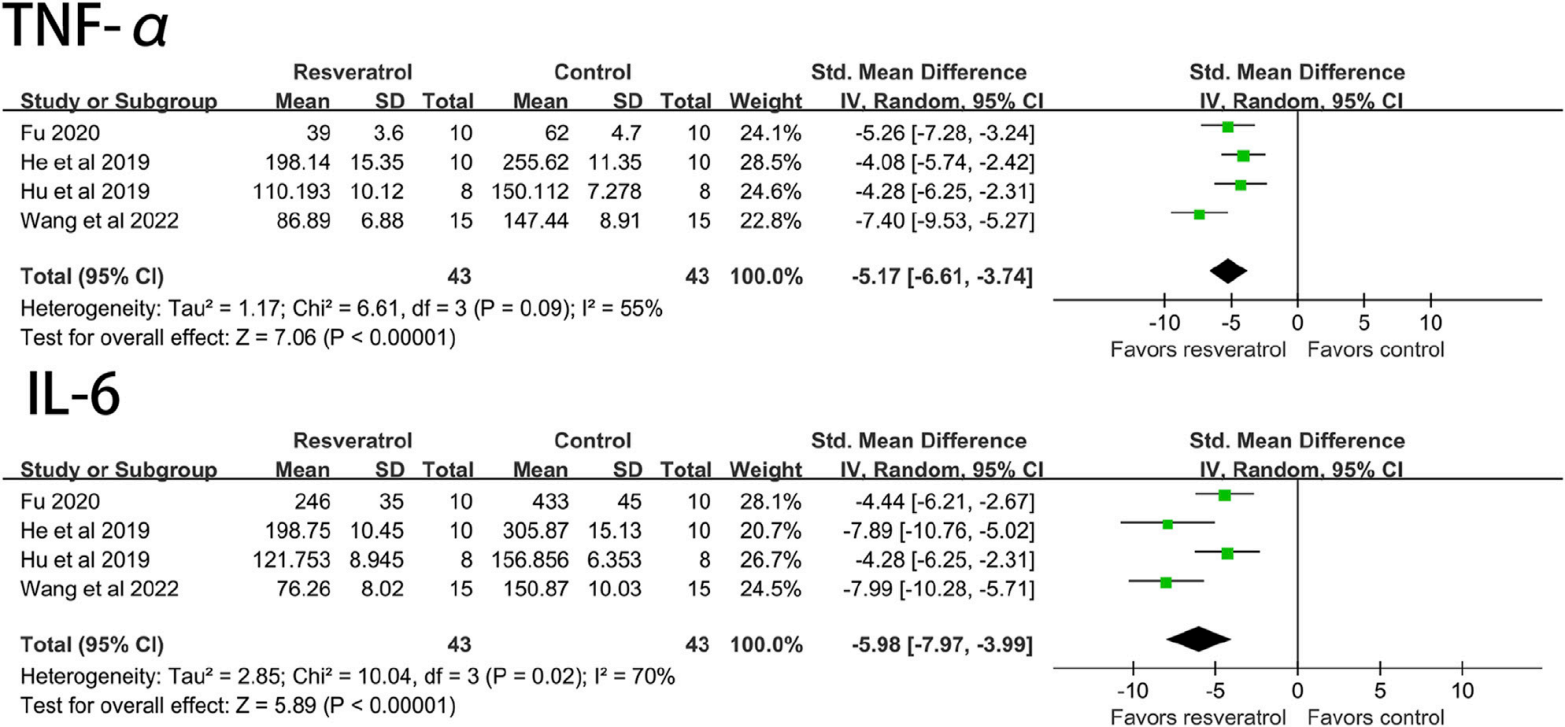
Forest plot of effects of resveratrol on TNF-a, IL-6 in rats with myocardial ischemia-reperfusion injury. TNF-a, tumor necrosis factor-a; IL-6, interleukin-6.

Studies have shown that resveratrol can significantly reduce TNF-α (four studies, 86 animal models, I^2^ = 55%, *p* < 0.05, SMD = −5.71 [−6.61, −3.74]) and IL-6 (four studies, 86 animal models, I^2^ = 70%, *p* < 0.05). SMD = −5.98 [−7.97, −3.99] level of the indicator. See Figure 5.

#### 3.3.5 Effect of resveratrol on apoptosis of myocardial ischemia-reperfusion injury model in rats

**FIGURE 6:**
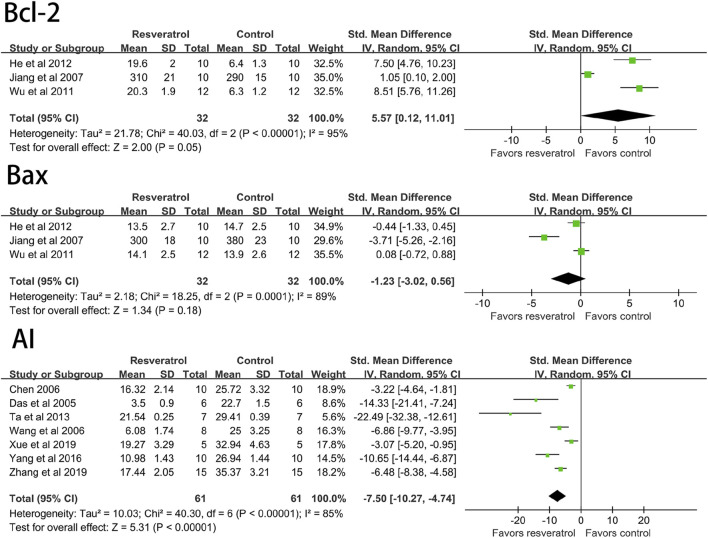
Forest plot of effects of resveratrol on Bcl-2, Bax, AI in rats with myocardial ischemia-reperfusion injury. Bcl-2, B-cell lymphoma-2; Bax, BCL2-Associated X; AI, cardiomyocyte apoptosis index.

The results of meta-analysis of forest maps showed that resveratrol could significantly reduce the apoptosis index of cardiomyocytes (seven studies, 122 animal models, I^2^ = 85%, *p* < 0.05, SMD = −7.50 [−10.27, −4.74]) and significantly increase Bcl-2 (three studies, 64 animal models, I^2^ = 95%, *p* < 0.05, SMD = 5.57 [0.12, 11.01]) level. However, there was no significant effect on Bax (three studies, 64 animal models, I^2^ = 89%, *p* = 0.18, SMD = 1.23 [−3.02, 0.56]. See Figure 6.

#### 3.3.6 Effects of resveratrol on cardiac tissue structure in rat model of myocardial ischemia-reperfusion injury

**FIGURE 7:**
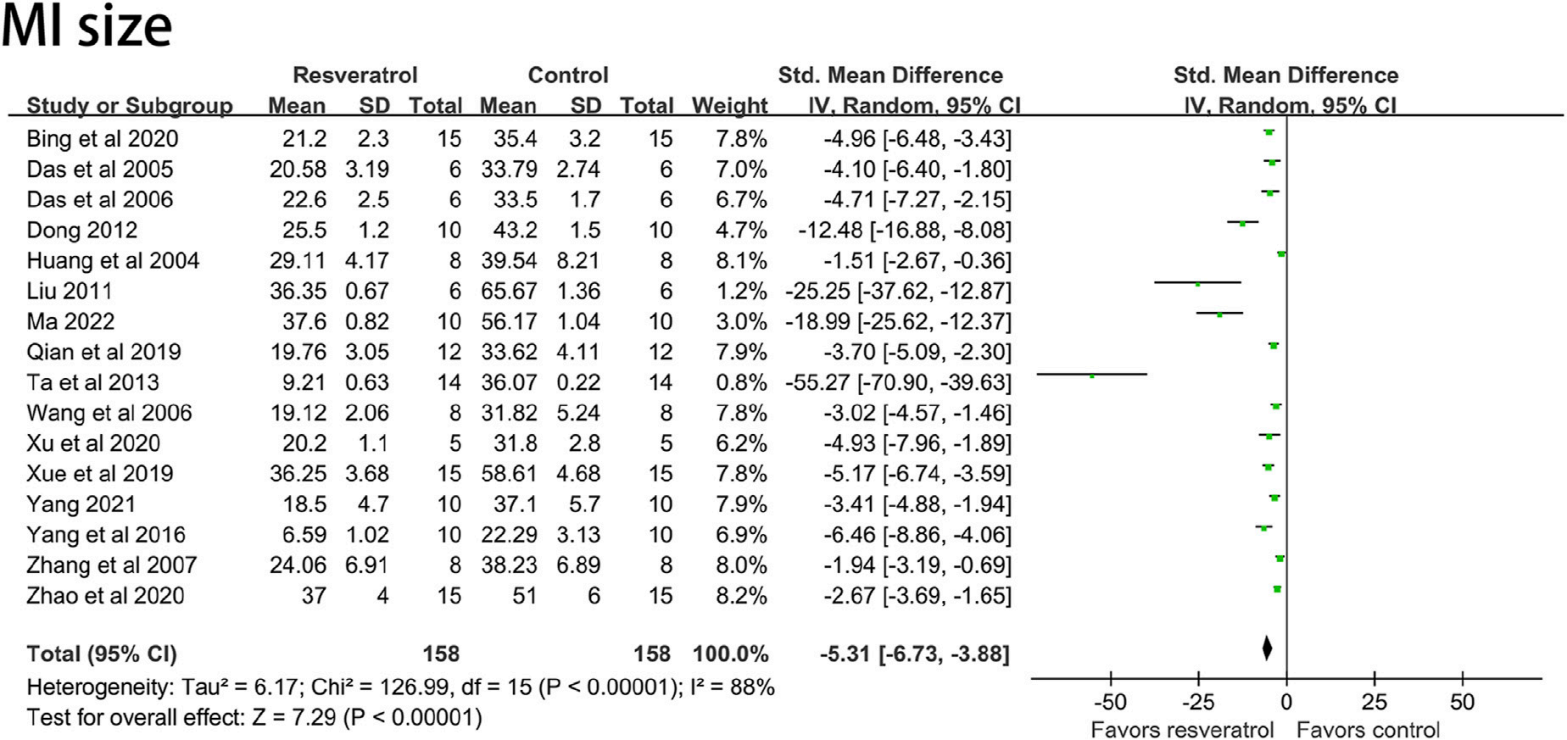
Forest plot of effects of resveratrol on MI size in rats with myocardial ischemia-reperfusion injury. MI size, myocardial infarction size.

Sixteen studies (296 animal models) quantitatively reported the change of myocardial infarction size, and there was significant statistical heterogeneity among the studies (I^2^ = 88%, *p* < 0.05, SMD = −5.31 [−6.77, −3.86]). The myocardial infarction size of the experimental group was significantly lower than that of the control group. See Figure 7.

#### 3.3.7 Subgroup analysis of routes of administration

In order to study the effects of different administration routes of resveratrol on rat models of myocardial ischemia-reperfusion injury, the literatures included under different outcome indexes were classified and integrated according to Intragastric administration (i.g), intraperitoneal injection (i.p), intravenous injection (i.v), and extracorporeal perfusion (ecp).

**FIGURE 8:**
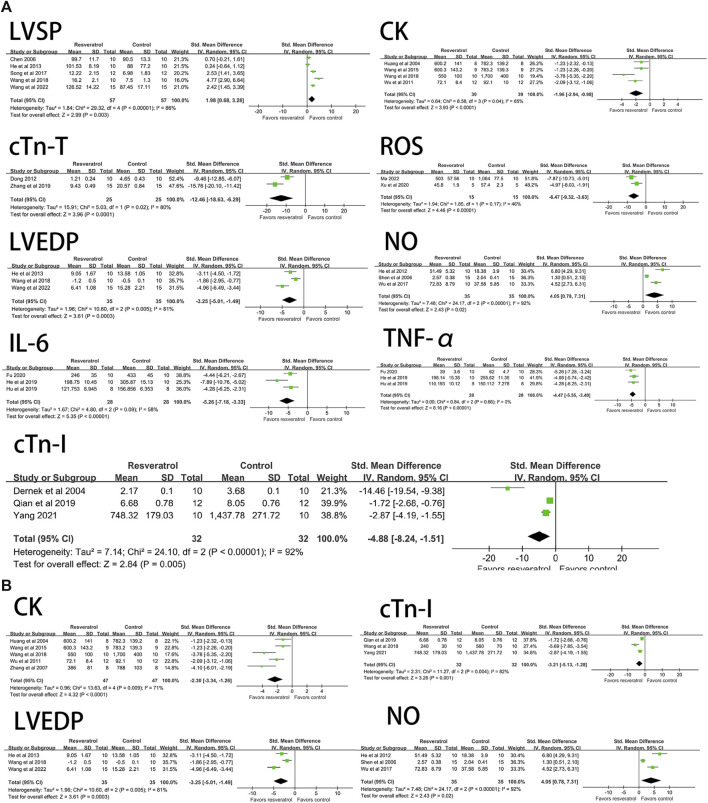
**(A)** Forest plot for subgroup analysis of administration route: the effects of resveratrol on LVSP, CK, cTn‐T, ROS, LVEDP, NO, IL‐6, TNF‐α, cTn‐I in rats with myocardial ischemia-reperfusion injury. **(B)** Forest plot for subgroup analysis of modeling mothod: the effects of resveratrol on LVEDP, CK, NO, cTn‐I in rats with myocardial ischemia-reperfusion injury. LVSP, left ventricular systolic pressure; CK, creatine kinase; cTn-T, cardiae troponin T; ROS, reactive oxygen species: LVEDP, left ventricular end-diastolic pressure; N0, nitric oxides; IL-6, interleukin-6; TNF-a, tumor necrosis factor-a; cTn-I, cardiac troponin I.

According to different routes of administration, there was only one study on intragastric and *in vitro* perfusion among LVSP (Dernek et al., 2004; Liu, 2011) and CK (Zhang et al., 2007; Wang et al., 2014) indexes. Among the indexes of IL-6 (Wang et al., 2022), TNF-α (Wang et al., 2022) and cTn-I (Wang and Zhang, 2018), there was only one study on intravenous injection. Among LVEDP (Wang et al., 2014) and NO (Wang et al., 2006) indexes, there was only one paper involving *in vitro* perfusion. There was only one study on the outcome indexes of cTn-T (Yang, 2021) and ROS (Yang, 2021). After deleting the literatures that appeared only once under the above indicators, we re-observed the influence of the remaining uniform drug administration routes on the study results. The results showed that LVSP (I^2^ = 86%, *p* < 0.05, SMD = 1.98 [0.68, 3.28]), CK (I^2^ = 65%, *p* < 0.05, SMD = −1.96 [−2.94, −0.98]), LVEDP (I^2^ = 81%, *p* < 0.05, SMD = −3.25 [−5.01, −1.49]), NO (I^2^ = 92%, *p* < 0.05, SMD = 4.05 [0.78, 7.31]), cTn-T (I^2^ = 80%, *p* < 0.05, SMD = −12.46 [−18.63, −6.29]), ROS (I^2^ = 46%, *p* < 0.05, SMD =−6.47 [-9.32, −3.63]), IL-6 (I^2^ = 58%, *p* < 0.05, SMD = −5.26 [−7.18, −3.33]), TNF-α (I^2^ = 0%, *p* < 0.05, SMD = −4.47 [−5.55, −3.40]), cTn-I (I^2^ = 92%, *p* < 0.05, SMD = −4.88 [−8.24, −1.51]) and the results were not significantly different before reference removal. The result is shown in Figure 8A.

**FIGURE 9:**
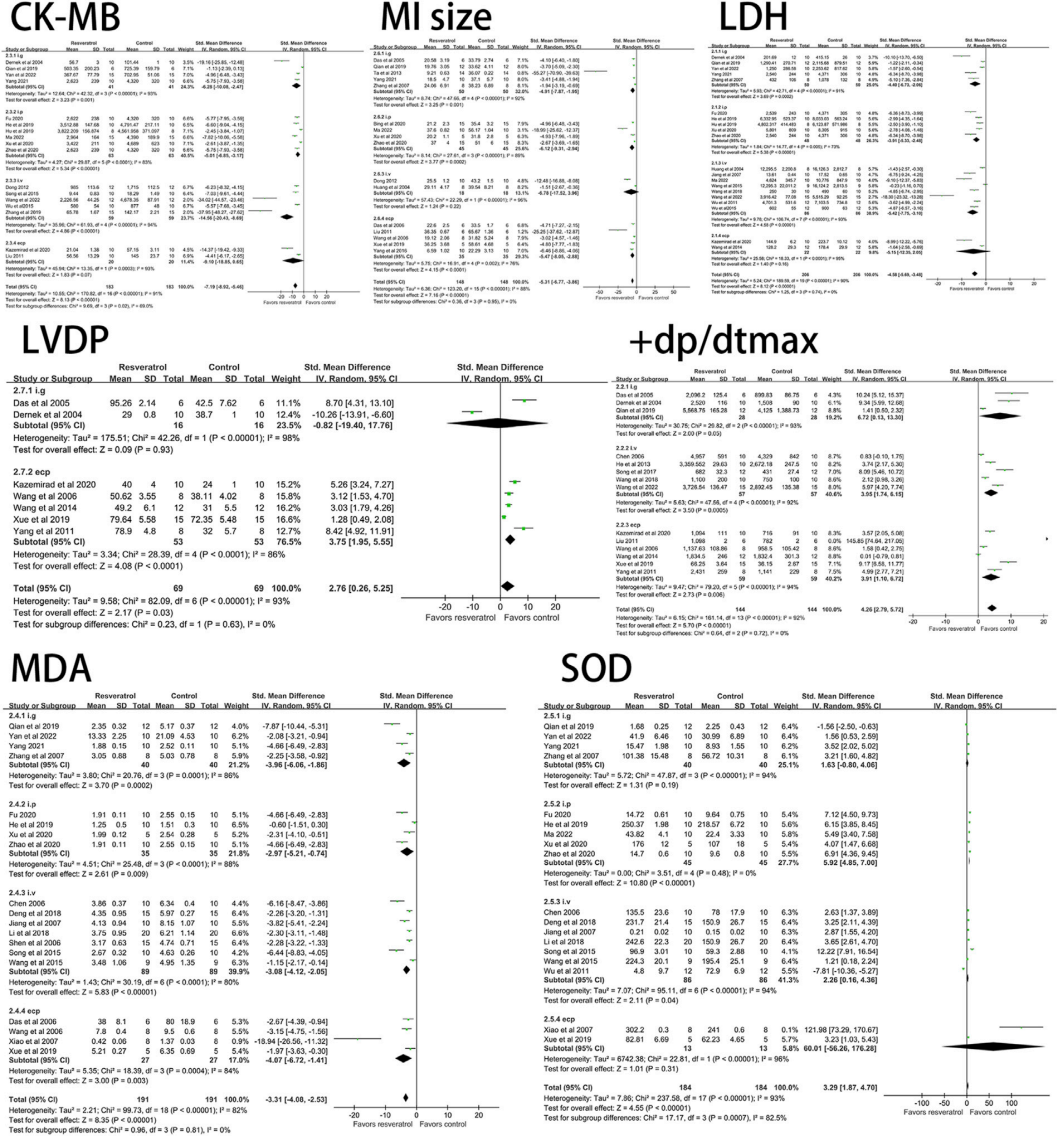
Forest plot for subgroup analysis of administration route: the effects of resveratrol on CK-MB, MI size, LVDP, LDH, MDA, SOD. +dp/dtmax in rats with myocardial ischemia-reperfusion injury. CK-MB, creatine kinase-MB: Mll size, myocardial infarction size; LVDP, left ventricular diastolic pressure:LDH, lactate dehydrogenase; MDA, malondialdehyde; S0D, superoxide dismtase; +dp/dtmax, maximun rate of Increase of left ventricular pressure.

Among the seven outcome indexes of +dp/dtmax, LDH, CK-MB, MDA, SOD, myocardial infarction size and LVDP, there were ≥ two drug administration pathways involved, and the number of research literatures for each pathway was ≥ two. According to the different routes of administration, the included literatures were re-classified and statistically analyzed. The result is shown in Figure 9. The results of subgroup analysis showed: Among the four subgroups under CK-MB and LDH outcome indexes, the *p* values of i.g, i.p and i.v administration route groups were all <0.05, resveratrol reduced CK-MB (five studies, SMD = −14.56 [−20.43, −8.69]) and LDH (eight studies, SMD = −5.42 [−7.75, −3.10]) levels best in the iv group compared to the ip and ig groups. However, the study results of ecp administration mode under CK-MB and LDH indexes were *p* = 0.07, SMD = −9.10 (−18.85, 0.65) and *p* = 0.16, SMD = −5.15 (−12.35, 2.05) respectively, indicated that this route of administration did not have a significant impact on the CK-MB and LDH indexes. In the four subgroup studies on myocardial infarction size, the *p* values of i.g, i.p and ecp subgroups were all <0.05, resveratrol reduced MI size (five studies, SMD = −6.12 [−9.31, −2.94]) level best in the ip group compared to the ecp and ig groups, however, *p* = 0.22 and SMD = −6.78 (−17.52, 3.96) under i.v administration pathway. In the SOD subgroup study, the results of i.p and i.v subgroups were *p* < 0.05, resveratrol increased the SOD level, ip group (five studies, SMD = 5.92 [4.85, 7.00]) was higher than iv group (seven studies, SMD = 2.26 [0.16, 4.36]), but the results of i.g and ecp subgroups were *p* = 0.19, SMD = 1.63 (−0.80, 4.06) and *p* = 0.31, SMD = 60.01 (−56.26, 176.28), respectively. In the subgroup study under LVDP index, the results of i.g group were *p* = 0.93 and SMD = −0.82 (−19.40, 17.76). The results of drug administration routes in the +dp/dtmax and MDA subgroups were all <0.05, these two indicators were not affected by the routes of administration. Under the MDA index, the resveratrol effect of ecp group was the best, but there was little difference compared with the other three groups, the ig group (three studies, SMD = 6.72 [0.13, 13.30]) elevated the +dp/dtmax level most significantly, compared with the other three groups. From the results of subgroup analysis, it can be seen that the drug effect of resveratrol was affected by the administration route.

#### 3.3.8 Subgroup analysis of modeling methods

The modeling methods of myocardial ischemia-reperfusion injury in rats were divided into *in vivo* heart ligation model and *in vitro* heart perfusion device model according to whether or not *in vitro*. It was represented by ligation of the left anterior descending branch (LAD) and Langendorff isolated heart perfusion. The electrical activity and circulatory function of the heart are affected during the *in vivo* ligation procedure. Isolated heart perfusion has few influencing factors because the heart is not regulated by nerves and body fluids. In order to study the effects of different modeling methods on the model of myocardial ischemia-reperfusion injury in rats, we classified and integrated the literatures included under different outcome indicators according to *in vivo* ligation and *in vitro* perfusion.

Classification according to different modeling methods shows that there is only one research paper on Langendorff model among LVEDP (Wang et al., 2014), CK (Wang et al., 2014), cTn-I (Dernek et al., 2004) and NO (Wang et al., 2006) indexes. After removing the literatures that appeared only once under the above indicators, we re-observed the influence of the remaining unified modeling method on the research results. The results showed that compared with the literatures before exclusion, the intervention effect of resveratrol on LVEDP (I^2^ = 81%, *p* < 0.05, SMD = −3.25 [-5.01, −1.49]), CK (I^2^ = 71%, *p* < 0.05, SMD = −2.30 [−3.34, −1.26]), NO (I^2^ = 92%, *p* < 0.05, SMD = 4.05 [0.78, 7.31]), cTn-I (I^2^ = 82%, *p* < 0.05, SMD = −3.21 [−5.13, −1.28]) indexes did not fluctuate significantly. The result is shown in Figure 8B.

**FIGURE 10:**
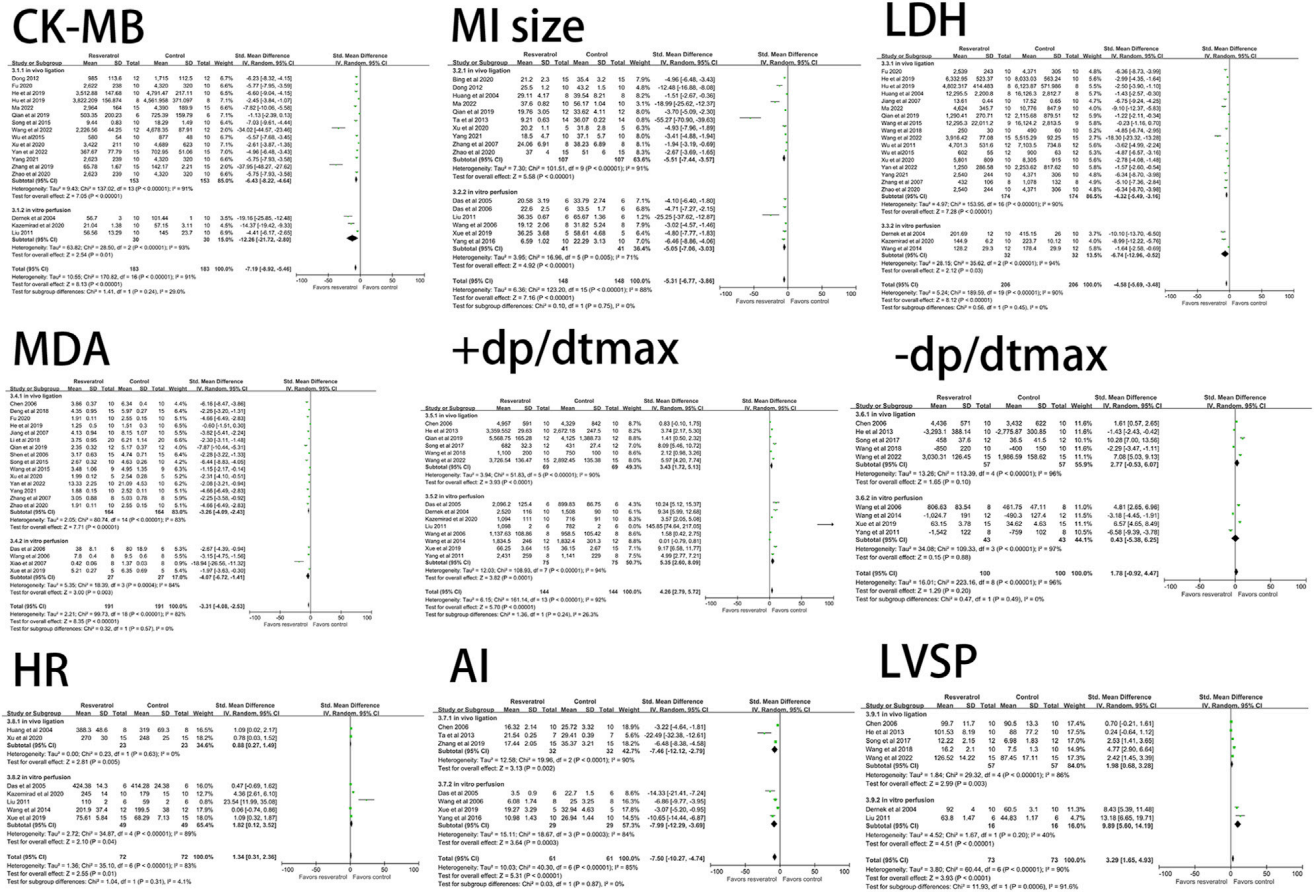
Forest plot for subgroup analysis of modeling method: the effects of resveratrol on CK-MB, MI size, LDH, MDA, AI, +dp/dtmax, -dp/dtmax, HR in rats with myocardial ischemia-reperfusion injury. CK-MB, creatine kinase-MB; MI size, myocardial infarction size; AI, cardiomyocyte apoptosis index; LDH, lactate dehydrogenase; MDA, malondialdehyde; HR, heart rate; +dp/dtmax, maximum rate of increase of left ventricular pressure; -dp/dtmax, maximum rate of decrease of left ventricular pressure; LVSP, left ventricular systolic pressure.

Among the research literatures on the nine outcome indicators of -dp/dtmax, +dp/dtmax, LDH, CK-MB, LVSP, MDA, AI, MI Size and HR, there were two modeling methods involved, and the number of research literatures on each approach was ≥ two. According to the different modeling methods, the classified statistics of the included literatures were re-carried out. The result is shown in Figure 10. Subgroup analysis results showed that: In the subgroups of *in vitro* perfusion and *in vivo* ligation, compared with the control group, resveratrol can increase + dp/dtmax, LVSP, HR levels, improve cardiac function, and the group vitro was higher than the vivo group. Resveratrol can reduce LDH, CK-MB, MDA, AI, MI Size levels, the effect was higher in the vitro group than in the vivo group in terms of LDH, CK-MB, MDA, and AI, on the contrary, the vivo group was higher than the vitro group under the MI Size index. However, under the -dp/dtmax outcome index, the results of *in vivo* ligation and *in vitro* perfusion subgroups were *p* = 0.10, SMD = 2.77 (−0.53, 6.07) and *p =* 0.87, SMD = 0.49 (−5.21, 6.18), respectively, indicated that resveratrol did not have a good therapeutic effect on the -dp/dtmax index, and this result was not affected by the model preparation method. Compared with before grouping, it can be seen that the model preparation method has little affect on the therapeutic effect of resveratrol.

## 4 Discussion

Animal experiments are the preliminary basis for clinical trials, through which the safety of intervention measures can be evaluated and the dose-effect relationship and effectiveness of drugs can be preliminaries judged, providing strong evidence for whether clinical treatment measures or new drugs can be put into clinical trials. In this meta-analysis, we selected a rat model of myocardial ischemia-reperfusion injury as the study object, aiming to exclude the influence of animal species differences on the study results. We found that resveratrol can reduce ST segment, cTn-I, cTn-T, CK, CK-MB, LDH, LVEDP, ROS, MDA, TNF-α, IL-6, AI levels and myocardial infarction size. HR, LVDP, LVSP, +dp/dtmax, NO, Bcl-2, and SOD levels were increased. However, resveratrol had no significant effect on -dp/dtmax and Bax outcome measures.

Studies (Kazemirad and Kazerani, 2020; Rodrigo et al., 2022; Li et al., 2023) have shown that oxidative stress is the main factor aggravating MIRI, and the production of a large number of oxygen free radicals can lead to lipid peroxidation of myocardial membrane, which is one of the important mechanisms of reperfusion injury. As an intermediate metabolite of lipid peroxides, MDA can destroy cell membrane structure, lead to changes in cell membrane fluidity and permeability, activation of cell membrane ion channels, myocardial electrophysiological changes, enzyme release in cardiomyocytes, and even failure to maintain normal metabolism of cells, resulting in arrhythmia and myocardial infarction. It is often used as an index to reflect the formation of oxygen free radicals and the damage caused by membrane. SOD is a necessary enzyme for scavenging oxygen free radicals in myocardium, and its activity reflects the degree of antioxidation in ischemia-reperfusion myocardium. NO can relax vascular smooth muscle, dilate coronary artery, reduce myocardial oxygen consumption and play a protective role in I/R injury. Reperfusion of cardiomyocytes can also cause excessive inflammation, release cytokines such as TNF-α and IL-6, and stimulate further myocardial damage (Yuan et al., 2018). Resveratrol can decrease MDA, TNF-α, IL-6 and increase SOD and NO levels, indicating that resveratrol can inhibit and protect oxidative damage and inflammation.

A review (Korshunova et al., 2021) by Korshunova: BCL2 regulates apoptosis in myocardial ischemia-reperfusion injury, discussed the important mechanism of apoptosis in myocardial ischemia/reperfusion injury. Apoptosis is controlled by many apoptosis-related genes. The Bcl-2 family, including bcl-2 and bax, is a major apoptotic regulatory gene. Among them, bcl-2 promoted cell survival and inhibited apoptosis, while bax promoted apoptosis. Resveratrol can reduce the apoptosis index, although the decrease of bax is not obvious, but significantly increase the level of Bcl-2.

cTn-I, cTn-T, CK, CK-MB, LDH are commonly used markers to evaluate myocardial injury and myocardial infarction, but there are differences in sensitivity and specificity. When myocardial ischemia and hypoxia lead to degeneration and necrosis, blood cTn-T and cTn-I begin to rise at four-12 h and last for four—10 days. cTn is regarded as the preferred marker of myocardial injury due to its long half-life, high content in myocardium and high sensitivity. In the study, resveratrol decreased the levels of cTn-I, cTn-T, CK, CK-MB, LDH, and alleviated myocardial damage.

Subgroup analysis was performed on CK-MB, myocardial infarction size, LDH, ROS, LVDP and other indicators according to the route of administration. The results showed that the route of administration had an impact on the heterogeneity of the study, which may be due to the differences in drug absorption and availability. However, this is not the only factor to investigate the source of heterogeneity, and may also be related to the dose, mode of modeling, ligation and reperfusion time. In this meta-analysis, several studies (literature) grouped the dose of resveratrol into low, medium, and high doses (Huang et al., 2004; Zhang et al., 2007; Huang et al., 2009; Dong, 2012; Yan et al., 2022). The results of these studies showed that resveratrol showed a gradient effect at different doses, and the high-dose group had the best therapeutic effect. We extracted the high-dose group data from these studies for statistical analysis. Due to the problems of unit disunity and large distribution difference in the dose of 43 literatures included in the study, subgroup analysis of the dose was not conducted for the time being.

This Meta-analysis was implemented based on means and standard deviations, pooled at the overall level of each study, due to the differences between individual study designs, the limitations of this study are inevitably exposed: only a few of the included studies described the properties (purity) of resveratrol, and the methodological quality of the studies was low, for example, the principles of “randomization” and “blind method” were not explained or adopted, which may lead to bias in the selection, measurement, implementation and reporting of the studies, resulting in biased research results; The intervention dose and administration time of each study group were not uniform, most of them were administered only once before surgery, and the long-term efficacy could not be determined; The dose-effect relationship between the experimental groups has not been deeply discussed, this makes it unknown whether the study results will vary greatly in terms of dose differences, resulting in the optimal therapeutic dose range of resveratrol being ignored, which will not only affect the dose selection of subsequent researchers, but also make the clinical trial of the drug lack an important grip.

## 5 Conclusion

Our current analysis results show that resveratrol can alleviate myocardial injury in rat myocardial ischemia-reperfusion injury model, improve left ventricular systolic and diastolic ability, alleviate inflammatory response, reduce myocardial cell necrosis and apoptosis, and reduce myocardial infarction size, and has a good therapeutic effect on MIRI. RES may be a promising drug to treat MIRI. However, the exact mechanism of action and clinical efficacy still need to be verified in animal experiments and clinical randomized controlled trials. Therefore, high-quality *in vivo* experiments in rats are still encouraged to investigate the protective effects of resveratrol on MIRI.

## Data Availability

The original contributions presented in the study are included in the article/Supplementary material, further inquiries can be directed to the corresponding author.
